# Supplementary material for: Fasting plasma glucose levels are associated with all-cause and cancer mortality: A population-based retrospective cohort study
Source: PLoS One. 2024 Nov 19;19(11):e0311150. doi: 10.1371/journal.pone.0311150 (PMC11575760; doi:10.1371/journal.pone.0311150)
Supplement: S1 Table — (DOCX) [file pone.0311150.s001.docx]

**S1 Table Basic demographic characteristics of the all-cause mortality group**

| Characteristic | Overall | All-cause mortality | | *P* value |
| --- | --- | --- | --- | --- |
|  |  | No | Yes |  |
| No. of cases | 148755 | 138410 | 10345 |  |
| Person-year (total) | 789150.10 | 757835.69 | 31314.41 |  |
| Follow-up time, years [M (P25, P75)] | 5.49 [5.31, 5.59] | 5.51 [5.35, 5.60] | 3.11 [1.77, 4.33] | <0.001 |
| Sex [n (%)] |  |  |  | <0.001 |
| Men | 64013 (43.0) | 57996 (41.9) | 6017 (58.2) |  |
| Women | 84742 (57.0) | 80414 (58.1) | 4328 (41.8) |  |
| Age, years [M (P25, P75)] | 60.00 [51.00, 68.00] | 59.00 [50.00, 67.00] | 77.00 [68.00, 82.00] | <0.001 |
| SBP, mmHg [M (P25, P75)] | 130.00 [120.00, 140.00] | 130.00 [120.00, 140.00] | 134.00 [124.00, 146.00] | <0.001 |
| DBP, mmHg [M (P25, P75)] | 79.00 [70.00, 84.00] | 79.00 [70.00, 84.00] | 79.00 [70.00, 84.00] | 0.704 |
| BMI, kg/m² [M (P25, P75)] | 22.87 [20.96, 24.97] | 22.94 [21.09, 25.02] | 21.77 [19.82, 23.90] | <0.001 |
| FPG, mmol/L [M (P25, P75)] | 5.40 [4.93, 6.00] | 5.40 [4.90, 6.00] | 5.60 [5.00, 6.23] | <0.001 |
| Cigarette smoking [n (%)] |  |  |  | <0.001 |
| Never smoking | 114881 (77.2) | 107650 (77.8) | 7231 (69.9) |  |
| Formerly smoking | 6582 (4.4) | 5733 (4.1) | 849 (8.2) |  |
| Current smoking | 27292 (18.3) | 25027 (18.1) | 2265 (21.9) |  |
| Physical exercise [n (%)] |  |  |  | <0.001 |
| Every day | 17824 (12.0) | 16824 (12.2) | 1000 (9.7) |  |
| More than once a week | 8328 (5.6) | 7893 (5.7) | 435 (4.2) |  |
| Occasionally | 13777 (9.3) | 13107 (9.5) | 670 (6.5) |  |
| Never | 108826 (73.2) | 100586 (72.7) | 8240 (79.7) |  |
| Alcohol consumption [n (%)] |  |  |  | <0.001 |
| Never drinking | 114290 (76.8) | 106519 (77.0) | 7771 (75.1) |  |
| Occasionally drinking | 9800 (6.6) | 9188 (6.6) | 612 (5.9) |  |
| Often drinking | 7723 (5.2) | 7115 (5.1) | 608 (5.9) |  |
| Every day | 16942 (11.4) | 15588 (11.3) | 1354 (13.1) |  |

Abbreviations: FPG, fasting plasma glucose; BMI, body mass index; SBP, systolic blood pressure; DBP, diastolic blood pressure
