# Supplementary material for: Fasting plasma glucose levels are associated with all-cause and cancer mortality: A population-based retrospective cohort study
Source: PLoS One. 2024 Nov 19;19(11):e0311150. doi: 10.1371/journal.pone.0311150 (PMC11575760; doi:10.1371/journal.pone.0311150)
Supplement: S2 Table — (DOCX) [file pone.0311150.s002.docx]

**S2 Table Basic demographic characteristics of the cancer mortality group**

| Characteristic | Overall | Cancer mortality | | *P* value |
| --- | --- | --- | --- | --- |
|  |  | No | Yes |  |
| No. of cases | 148755 | 145910 | 2845 |  |
| Person-year (total) | 789150.10 | 780594.63 | 8555.46 |  |
| Follow-up time, years [M (P25, P75)] | 5.49 [5.31, 5.59] | 5.50 [5.32, 5.59] | 3.06 [1.67, 4.33] | <0.001 |
| Sex [n (%)] |  |  |  | <0.001 |
| Men | 64013 (43.0) | 62154 (42.6) | 1859 (65.3) |  |
| Women | 84742 (57.0) | 83756 (57.4) | 986 (34.7) |  |
| Age, years [M (P25, P75)] | 60.00 [51.00, 68.00] | 60.00 [51.00, 68.00] | 70.00 [63.00, 78.00] | <0.001 |
| SBP, mmHg [M (P25, P75)] | 130.00 [120.00, 140.00] | 130.00 [120.00, 140.00] | 132.00 [122.00, 144.00] | <0.001 |
| DBP, mmHg [M (P25, P75)] | 79.00 [70.00, 84.00] | 79.00 [70.00, 84.00] | 78.00 [70.00, 84.00] | 0.064 |
| BMI, kg/m² [M (P25, P75)] | 22.87 [20.96, 24.97] | 22.89 [21.00, 24.98] | 22.14 [20.20, 24.26] | <0.001 |
| FPG, mmol/L [M (P25, P75)] | 5.40 [4.93, 6.00] | 5.40 [4.92, 6.00] | 5.50 [5.00, 6.10] | <0.001 |
| Cigarette smoking [n (%)] |  |  |  | <0.001 |
| Never smoking | 114881 (77.2) | 113102 (77.5) | 1779 (62.5) |  |
| Formerly smoking | 6582 (4.4) | 6324 (4.3) | 258 (9.1) |  |
| Current smoking | 27292 (18.3) | 26484 (18.2) | 808 (28.4) |  |
| Physical exercise [n (%)] |  |  |  | <0.001 |
| Every day | 17824 (12.0) | 17513 (12.0) | 311 (10.9) |  |
| More than once a week | 8328 (5.6) | 8205 (5.6) | 123 (4.3) |  |
| Occasionally | 13777 (9.3) | 13596 (9.3) | 181 (6.4) |  |
| Never | 108826 (73.2) | 106596 (73.1) | 2230 (78.4) |  |
| Alcohol consumption [n (%)] |  |  |  | <0.001 |
| Never drinking | 114290 (76.8) | 112306 (77.0) | 1984 (69.7) |  |
| Occasionally drinking | 9800 (6.6) | 9637 (6.6) | 163 (5.7) |  |
| Often drinking | 7723 (5.2) | 7535 (5.2) | 188 (6.6) |  |
| Every day | 16942 (11.4) | 16432 (11.3) | 510 (17.9) |  |

Abbreviations: FPG, fasting plasma glucose; BMI, body mass index; SBP, systolic blood pressure; DBP, diastolic blood pressure
