# Supplementary material for: Fasting plasma glucose levels are associated with all-cause and cancer mortality: A population-based retrospective cohort study
Source: PLoS One. 2024 Nov 19;19(11):e0311150. doi: 10.1371/journal.pone.0311150 (PMC11575760; doi:10.1371/journal.pone.0311150)
Supplement: S3 Table — (DOCX) [file pone.0311150.s003.docx]

**S3 Table Subgroup analyses of the association between the FPG and risk of mortality**

|  |  | Model | Fasting Glucose Categories | | | |
| --- | --- | --- | --- | --- | --- | --- |
|  |  | HR (95%CI) | LFG | NFG | IFG | DM |
| All-cause mortality | Men | Model 1 | 1.33 (1.11,1.59) * | 1.00 (REF) | 1.28 (1.21,1.36) ** | 1.75 (1.62,1.89) ** |
|  |  | Model 2 | 1.21 (1.01,1.44) * | 1.00 (REF) | 1.06 (1.00,1.12) * | 1.37 (1.27,1.48) ** |
|  |  | Model 3 | 1.14 (0.95,1.36) | 1.00 (REF) | 1.08(1.03,1.15) * | 1.40 (1.29,1.51) ** |
|  | Women | Model 1 | 1.27 (1.00,1.61) * | 1.00 (REF) | 1.42 (1.33,1.52) ** | 1.93 (1.77,2.10) ** |
|  |  | Model 2 | 1.20 (0.94,1.52) | 1.00 (REF) | 1.13 (1.05,1.20) ** | 1.47 (1.34,1.60) ** |
|  |  | Model 3 | 1.13(0.89,1.44) | 1.00 (REF) | 1.14 (1.07,1.22) ** | 1.50(1.38,1.64) ** |
|  | <60 years | Model 1 | 1.37 (0.85,2.19) | 1.00 (REF) | 1.36 (1.18,1.57) ** | 2.44 (2.04,2.91) ** |
|  |  | Model 2 | 1.38 (0.86,2.20) | 1.00 (REF) | 1.39 (1.21,1.61) ** | 2.52 (2.12,3.01) ** |
|  |  | Model 3 | 1.35 (0.84,2.16) | 1.00 (REF) | 1.36 (1.18,1.57) ** | 2.41 (2.01,2.88) ** |
|  | ≥60 years | Model 1 | 1.21 (1.04,1.41) * | 1.00 (REF) | 1.15 (1.10,1.20) ** | 1.44 (1.35,1.53) ** |
|  |  | Model 2 | 1.19 (1.02,1.38) * | 1.00 (REF) | 1.17 (1.12,1.22) ** | 1.49 (1.40,1.58) ** |
|  |  | Model 3 | 1.16 (0.99,1.34) | 1.00 (REF) | 1.21 (1.15,1.26) ** | 1.53 (1.44,1.63) ** |
| Cancer mortality | Men | Model 1 | 1.31 (0.96,1.79) | 1.00 (REF) | 1.18 (1.07,1.31) ** | 1.44 (1.24,1.66) ** |
|  |  | Model 2 | 1.20 (0.87,1.64) | 1.00 (REF) | 1.02 (0.92,1.12) | 1.18 (1.02,1.36) * |
|  |  | Model 3 | 1.14 (0.83,1.56) | 1.00 (REF) | 1.05 (0.95,1.16) | 1.20 (1.04,1.39) * |
|  | Women | Model 1 | 1.40 (0.88,2.21) | 1.00 (REF) | 1.25 (1.09,1.44) * | 1.56 (1.29,1.88) ** |
|  |  | Model 2 | 1.35 (0.86,2.14) | 1.00 (REF) | 1.05(0.92,1.21) | 1.24 (1.03,1.50) * |
|  |  | Model 3 | 1.33 (0.84,2.10) | 1.00 (REF) | 1.06 (0.92,1.21) | 1.26 (1.04,1.52) * |
|  | <60 years | Model 1 | 1.03 (0.46,2.32) | 1.00 (REF) | 1.30 (1.05,1.61) * | 1.84 (1.37,2.47) ** |
|  |  | Model 2 | 1.03 (0.46,2.33) | 1.00 (REF) | 1.33 (1.07,1.64) * | 1.90 (1.41,2.54) ** |
|  |  | Model 3 | 1.02 (0.45,2.29) | 1.00 (REF) | 1.31 (1.06,1.63) * | 1.84 (1.37,2.48) ** |
|  | ≥60 years | Model 1 | 1.31 (0.99,1.72) | 1.00 (REF) | 1.03 (0.94,1.12) | 1.15 (1.02,1.31) * |
|  |  | Model 2 | 1.26 (0.96,1.66) | 1.00 (REF) | 1.06 (0.97,1.15) | 1.23 (1.08,1.39) * |
|  |  | Model 3 | 1.24 (0.94,1.63) | 1.00 (REF) | 1.08 (0.99,1.18) | 1.25 (1.10,1.41) ** |

**P*<0.05, ***P*<0.001

Model 1: unadjusted

Model 2: adjusted for age, sex

Model 3: adjusted for SBP, DBP, BMI, physical exercise, smoking, and alcohol consumption on the basis of the Model 2

Abbreviations: LFG, low fasting glucose; NFG, normal fasting glucose; IFG, impaired fasting glucose; DM, diabetes mellitus
