# Supplementary material for: Fasting plasma glucose levels are associated with all-cause and cancer mortality: A population-based retrospective cohort study
Source: PLoS One. 2024 Nov 19;19(11):e0311150. doi: 10.1371/journal.pone.0311150 (PMC11575760; doi:10.1371/journal.pone.0311150)
Supplement: S4 Table — (DOCX) [file pone.0311150.s004.docx]

**S4 Table Sensitivity analysis of different FPG levels associated with all-cause mortality**

| Model  HR (95%CI) | Fasting Glucose Categories | | | |
| --- | --- | --- | --- | --- |
|  | LFG | NFG | IFG | DM |
| Exclusion of patients with cancer at baseline who died within one year of follow-up | | | | |
| Model 1 | 1.29 (1.10,1.51) * | 1.00 (REF) | 1.32 (1.27,1.39) ** | 1.77 (1.67,1.88) ** |
| Model 2 | 1.17 (1.00,1.37) * | 1.00 (REF) | 1.09 (1.04,1.15) ** | 1.40 (1.32,1.49) ** |
| Model 3 | 1.10 (0.94,1.29) | 1.00 (REF) | 1.12 (1.07,1.17) ** | 1.42 (1.33,1.51) ** |
| Exclusion of patients with cancer at baseline, death within one year of follow-up, and smoking | | | | |
| Model 1 | 1.23 (1.01,1.49) * | 1.00 (REF) | 1.37 (1.30,1.44) ** | 1.79 (1.66,1.92) ** |
| Model 2 | 1.11 (0.92,1.35) | 1.00 (REF) | 1.10 (1.04,1.16) ** | 1.40 (1.30,1.50) ** |
| Model 3 | 1.03 (0.85,1.24) | 1.00 (REF) | 1.12 (1.06,1.18) ** | 1.41 (1.31,1.51) ** |

**P*<0.05, ***P*<0.001

Model 1: unadjusted

Model 2: adjusted for age, sex

Model 3: adjusted for SBP, DBP, BMI, physical exercise, smoking, and alcohol consumption on the basis of the Model 2

Abbreviations: LFG, low fasting glucose; NFG, normal fasting glucose; IFG, impaired fasting glucose; DM, diabetes mellitus
