# Supplementary material for: Fasting plasma glucose levels are associated with all-cause and cancer mortality: A population-based retrospective cohort study
Source: PLoS One. 2024 Nov 19;19(11):e0311150. doi: 10.1371/journal.pone.0311150 (PMC11575760; doi:10.1371/journal.pone.0311150)
Supplement: S5 Table — (DOCX) [file pone.0311150.s005.docx]

**S5 Table Sensitivity analysis of the association of different FPG levels with cancer mortality**

| Model  HR (95%CI) | Fasting Glucose Categories | | | |
| --- | --- | --- | --- | --- |
|  | LFG | NFG | IFG | DM |
| Exclusion of patients with cancer at baseline who died within one year of follow-up | | | | |
| Model 1 | 1.40 (1.05,1.87) * | 1.00 (REF) | 1.17 (1.07,1.29) ** | 1.39 (1.22,1.59) ** |
| Model 2 | 1.27 (0.95,1.69) | 1.00 (REF) | 1.02 (0.94,1.12) | 1.18 (1.04,1.35) * |
| Model 3 | 1.21 (0.91,1.62) | 1.00 (REF) | 1.04 (0.95,1.14) | 1.18 (1.03,1.34) * |
| Exclusion of patients with cancer at baseline, death within one year of follow-up, and smoking | | | | |
| Model 1 | 1.25 (0.85,1.83) | 1.00 (REF) | 1.21 (1.08,1.35) ** | 1.43 (1.22,1.67) ** |
| Model 2 | 1.15 (0.78,1.69) | 1.00 (REF) | 1.03 (0.92,1.15) | 1.19 (1.01,1.39) * |
| Model 3 | 1.11 (0.76,1.63) | 1.00 (REF) | 1.04 (0.93,1.16) | 1.17 (0.99,1.37) |

**P*<0.05, ***P*<0.001

Model 1: unadjusted

Model 2: adjusted for age, sex

Model 3: adjusted for SBP, DBP, BMI, physical exercise, smoking, and alcohol consumption on the basis of the Model 2

Abbreviations: LFG, low fasting glucose; NFG, normal fasting glucose; IFG, impaired fasting glucose; DM, diabetes mellitus
