# Supplementary material for: Fasting plasma glucose levels are associated with all-cause and cancer mortality: A population-based retrospective cohort study
Source: PLoS One. 2024 Nov 19;19(11):e0311150. doi: 10.1371/journal.pone.0311150 (PMC11575760; doi:10.1371/journal.pone.0311150)
Supplement: Attachment — Submitted filename: Response to Reviewers.docx [file pone.0311150.s006.docx]

Response to Reviewers

*Dear Editors and Reviewers:*

Thank you for your letter and for the reviewers’ comments concerning our manuscript entitled “**Fasting plasma glucose levels are associated with all-cause mortality and cancer death: a population-based retrospective cohort study**” (ID: PONE-D-23-43491). Those comments are all valuable and very helpful for revising and improving our paper, as well as the important guiding significance to our researches.

We have studied comments carefully and have made correction which we hope meet with approval. *PLOS ONE* is an influential journal. From all the papers published in your journal, readers have been learning a lot. Hopefully, we could have our article been considered of publication in your journal.

**Manuscript revision notes**: Revised portion are marked in red in the paper and deletions are in blue with strikethroughs.

***Response to*** ***academic editor***

***1. Please ensure that your manuscript meets PLOS ONE's style requirements, including those for file naming.***

We have reviewed our paper according to PLOS ONE's style requirements to ensure compliance with the journal's standards.

***2. In the online submission form, you indicated that "The data sets generated and/or analyzed during the current study are not publicly available due individual privacy information protection but are available from the corresponding author on reasonable request."***

***All PLOS journals now require all data underlying the findings described in their manuscript to be freely available to other researchers, either 1. In a public repository, 2. Within the manuscript itself, or 3. Uploaded as supplementary information. This policy applies to all data except where public deposition would breach compliance with the protocol approved by your research ethics board. If your data cannot be made publicly available for ethical or legal reasons (e.g., public availability would compromise patient privacy), please explain your reasons on resubmission and your exemption request will be escalated for approval.***

After careful consideration, we have confirmed that making the data publicly available would violate the protocol approved by our ethics committee and may pose a risk of privacy breaches. Therefore, we have provided an alternative method for data access. Interested researchers may request access to the data through the Ethics Review Committee of Quzhou Center for Disease Control and Prevention, subject to approval. We kindly ask the editorial board to consider our special circumstances.

**3. *When completing the data availability statement of the submission form, you indicated that you will make your data available on acceptance. We strongly recommend all authors decide on a data sharing plan before acceptance, as the process can be lengthy and hold up publication timelines. Please note that, though access restrictions are acceptable now, your entire data will need to be made freely accessible if your manuscript is accepted for publication. This policy applies to all data except where public deposition would breach compliance with the protocol approved by your research ethics board. If you are unable to adhere to our open data policy, please kindly revise your statement to explain your reasoning and we will seek the editor's input on an exemption. Please be assured that, once you have provided your new statement, the assessment of your exemption will not hold up the peer review process.***

After careful consideration, we have confirmed that making the data publicly available would violate the protocol approved by our ethics committee and may pose a risk of privacy breaches. Therefore, we have provided an alternative method for data access. Interested researchers may request access to the data through the Ethics Review Committee of Quzhou Center for Disease Control and Prevention, subject to approval. We kindly ask the editorial board to consider our special circumstances.

**4. *PLOS requires an ORCID iD for the corresponding author in Editorial Manager on papers submitted after December 6th, 2016. Please ensure that you have an ORCID iD and that it is validated in Editorial Manager.***

We have updated the corresponding author’s information and added an ORCID ID.

**5. *Your ethics statement should only appear in the Methods section of your manuscript. If your ethics statement is written in any section besides the Methods, please move it to the Methods section and delete it from any other section. Please ensure that your ethics statement is included in your manuscript, as the ethics statement entered into the online submission form will not be published alongside your manuscript.***

We have moved the ethics statement to the “Methods” section and deleted it from other sections.

***Reviewer 1***

Thanks very much for taking your time to review this manuscript. We are very grateful to your generous comments for the manuscript. I will answer your comments one by one：

**Response to comment:**

***This article is clearly structured, has some research significance, and the sample size is sufficient. The following are some problems that may need to be revised.***

***1.The 2 in the unit of BMI in line 91 should be superscripted***

Thank you for your reminder, I have corrected this error.

***2.Use a unified term for fasting plasma glucose and use abbreviations FPG, in line 152 and other lines***

According to your suggestion, I checked the entire manuscript and standardized the terminology for fasting plasma glucose, using the abbreviation FPG throughout. I also checked the usage of other terms in the article and standardized "all-cause and cancer death" to "all-cause and cancer mortality."

***3.Please describe the data screening process in detail and how many people were excluded***

I created a flowchart to illustrate the data screening process and the reasons and number of exclusions, making the analysis in the article clearer and more readable.

***4.Please conduct subgroup analysis by sex and age***

Thank you very much for your suggestion. I have added this part of the analysis, conducting subgroup analysis by age (< 60 years and ≥ 60 years) and gender to explore potential modifying effects. The subgroup analysis found that in different genders, the risk of all-cause death in the IFG and DM groups and the risk of cancer death in the DM group increased to varying degrees, but the risk of death in women was slightly higher than in men. The risk of hyperglycemia on all-cause mortality and cancer mortality was more pronounced in the <60 years age group.

***Reviewer 2***

We would like to thank the reviewers from the bottom of our hearts for carefully reviewing and guiding the paper, which has led to a great improvement in the quality of the paper.

**Response to comment:**

***#3 the data have not been made fully available, but the authors indicate "The data sets generated and/or analyzed during the current study are not publicly available due individual privacy information protection but are available from the corresponding author on reasonable request." This seems reasonable to me.***

***#4 the article is in good shape, but would benefit from English language review. I have pointed out some issues below.***

***1.The authors state that “the requirement for informed consent was waived due to the retrospective design.” This does not make sense to me. If the data come from human subjects, whether prospective or retrospective, the individuals should have the opportunity to provide informed consent or to request that their data not be used.***

This database comes from the 2015 resident health check-up data, which is part of a routine public health program. And in the retrospective analysis, we removed identifiable information, so the Quzhou CDC Ethics Review Committee waived the requirement for informed consent.

***2.Line 46 – what does this phrase refer to? “approximately 4.2 million adults aged 20 to 79 years [7].”***

This sentence refers to the number of adults aged 20 to 79 who died from diabetes in 2019. I have revised this sentence to make the content clearer

***3.Lines 50-54 – in regard to the study from Korea, the study examined the relationship between glucose levels and mortality in individuals with prevalent diabetes, and it appears that the risks were highest in those with the highest glucose levels compared with 85–99 mg/dL (not compared with the lowest levels of glucose). [I did not check the results of the other papers cited.]***

Thank you for your help. We apologize for our carelessness. Upon careful reading, it was found that in this prospective study in Korea, the relationship between fasting blood glucose and mortality in diabetic patients was examined. It was found that compared with 85-99 mg/dL, the multivariable adjusted risk ratio for hypoglycemia (<65 mg/dL) was 1.46. Avoiding hypoglycemia can improve the survival rate of diabetic patients. So we have revised it to "A prospective cohort study in Korea on the relationship between diabetic patients and mortality found that fasting blood glucose and all-cause mortality showed a U-shaped relationship, and hypoglycemia could increase the mortality rate of diabetic patients."

***4.Lines 67-69 - did this study include all residents of Quzhou City in that time period? If not, how were the individuals selected?***

Thank you for your question. This study did not include all residents of Quzhou City during that time period. Instead, it included individuals who voluntarily participated in the 2015 health check-ups organized by the Quzhou City Health Bureau. These health check-ups were part of a routine public health initiative, and participation was open to all residents. Participants were not randomly selected; however, the program was widely publicized and accessible to all segments of the population, which helps to ensure a diverse and representative sample of the community. Given the large sample size and the inclusivity of the health check-up program, we believe the cohort provides a reasonably representative cross-section of the Quzhou City population.

***5.Lines 71-73 need to be edited for clarity***

We have re-edited this content, and we want to express that the information in this database was entered into the Resident Health Check Information Database after quality control of the original data by trained professionals. This makes it easier for readers to understand the content.

***6.Line 83-84 – what about former smokers who had quit less than six months ago, how are they categorized?***

We categorized smoking status into three groups: never smoked, former smoker, and current smoker. Individuals who had previously smoked but quit for more than six months were classified as former smokers. If they had quit for less than six months, they were judged on the basis of their past smoking habits—if they had smoked at least 100 cigarettes, they were considered current smokers; otherwise, they were classified as never smoked. To avoid ambiguity, I changed 'current smoking' to 'smoking' for clearer expression.

***7.Line 90 – what were the “rigorous procedures” for collecting height and weight?***

We replaced the phrase "rigorous procedures" with specific content, referring to the fact that the height and weight of all participants were measured using standard instruments while they were wearing light clothing and had removed their shoes and hats.

***8.Line 104 – “lack of information on variables” is vague. What information was considered necessary in order for the individual not to be excluded?***

The lack of variable information refers to the lack of records on smoking, drinking, and physical exercise. In the article, we have clarified this to avoid ambiguity and confusion for readers.

***9.Table 1 – I think the all-cause mortality and the cancer mortality numbers represent those who are still alive (I had assumed they represented those who had died, but the numbers don’t make sense that way.) Please clarify.***

We apologize for the mistake. All-cause mortality and cancer mortality represent those who have died. We have corrected this part.

Based on reviewers' comments, we have revised the manuscript extensively. If there are any other modifications we could make, we would like very much to modify them and we really appreciate your help. We hope that our manuscript could be considered for publication in your journal. Thank you very much for your help.
